# Transactions of the Medical Society of the State of Alabama

**Published:** 1888-12

**Authors:** 


TRANSACTIONS OF THE MEDICAL SOCIETY OF THE STATE OF ALABAMA. Published by the Society. 1888.

We are indebted to the Secretary, Dr. Thos. A. Means, for this handsome volume of 410 pages.

It is a good index of the character of the most progressive and influential State society in the South.

The proceedings are of interest; the papers of great merit. As a whole, it is just what might be expected of such an association—very good.

